# Ectopic expression of *TTP* gene from human in poplar promotes xylem differentiation and confers plant drought tolerance

**DOI:** 10.48130/forres-0024-0008

**Published:** 2024-04-09

**Authors:** Yamei Zhuang, Yang Chen, Qiao Wang, Yan Chen, Liping Yan, Shengjun Li, Gongke Zhou, Guohua Chai

**Affiliations:** 1 College of Resources and Environment, Qingdao Agricultural University, Qingdao 266109, China; 2 Key Laboratory of Biofuels, Shandong Provincial Key Laboratory of Energy Genetics, Shandong Energy Institute, Qingdao New Energy Shangdong Laboratory, Qingdao Institute of Bioenergy and Bioprocess Technology, Chinese Academy of Sciences, Qingdao 266101, China; 3 Academy of Dongying Efficient Agricultural Technology and Industry on Saline and Alkaline Land in Collaboration with Qingdao Agricultural University, Dongying 257000, China; 4 College of Landscape Architecture and Forestry, Qingdao Agricultural University, Qingdao 266109, China; 5 Shandong Provincial Academy of Forestry, Jinan 250014, China

**Keywords:** CCCH protein, hTTP, Drought stress, Vessel cell, ROS scavenging activity, *Populus*

## Abstract

The CCCH zinc finger proteins play critical roles in a wide variety of growth, development, and stress responses. Currently, limited reports are available about the roles of animal CCCH proteins in plants. In this study, we report the identification of human TTP (hTTP) with functional similarity to PdC3H17 in a hybrid poplar. hTTP and PdC3H17 shared highly similar tandem CCCH zinc-finger RNA-binding domains. The fragments excluding the CCCH domain of both hTTP and PdC3H17 possessed transcriptional activation activities in yeast cells. Compared to the controls, ectopic expression of *hTTP* in poplar caused dwarfism, and resulted in significant increases in stem xylem vessel number and photosynthetic and ROS-scavenging abilities, thereby enhancing plant tolerance to drought stress. Our results suggest that hTTP may perform a function in poplar through the PdC3H17-mediated system, and provide an example for the application of animal genes in plants.

## Introduction

The CCCH zinc finger family contains one or several CCCH-type motif(s) and members of this family have been identified in many eukaryotic organisms from yeast to mammals^[1]^. In animals, the best known CCCH protein is human tristetraprolin (hTTP/ZFP36), which belongs to typical tandem CCCH Zinc Finger proteins (TZFs) and participates in multiple cell processes, including cell proliferation, metabolic homeostasis, autophagy, cancer and immune responses^[2]^. Mechanically, hTTP often binds to AU-rich elements (AREs) in the 3' UTR of target mRNAs and mediates the destabilization and degradation of mRNAs *via* RNA decay in the cytoplasm^[3]^. Also, TTP functions as a transcriptional co-repressor of multiple regulatory factors, such as NF-κB and steroid nuclear hormone receptors, in the cell nucleus^[4]^ .

Plant TZF proteins control a wide range of developmental and adaptive processes through protein–DNA, protein–RNA and protein–protein interactions, similar to the regulatory mechanisms of animal TZFs^[5,6]^. hTTP has two homologous proteins (AtC3H14 and AtC3H15) in Arabidopsis and three homologs (PdC3H17, PdC3H18 and PdC3H20) in poplar^[7]^. Arabidopsis AtC3H14 and AtC3H15 have both RNA- and DNA-binding capacities, and they redundantly regulate cell elongation and secondary cell wall (SCW) thickening in stem, fertility, and adaptive responses^[8−13]^. Similarly, poplar PdC3H17 regulates stem development during secondary growth and drought stress response^[14−17]^. At the molecular level, PdC3H17 positively regulates SCW deposition in stems and functions as a component of the PdMYB3/21-mediated pathway^[14]^. PdMYB3 and PdMYB21 are considered as second-level master switches of the transcription network for SCW deposition, and they directly activate the expression of *PdC3H17*^[14,18]^. PdC3H17 also directly activates the expression of *PdSAG101a* to modulate xylem cell differentiation^[16]^. In addition, PdC3H17 and PdMYB199 form an auxin-responsive functional complex, coordinately controlling cambium division by a dual mechanism^[15]^. Upon auxin treatment, *PdC3H17* transcription is enhanced, attenuating PdMYB199-driven suppression of cell division-associated target gene expression. The enhanced interaction between PaC3H17 and PdMYB199 further promotes the transcriptional repression of *PdMYB199* expression. Currently, it is unclear whether animal CCCH proteins execute functions in plants.

In this study, we provided evidence showing that hTTP protein may function in a way similar to PdC3H17 in a hybrid poplar. Transgenic poplar lines overexpressing *hTTP* exhibited morphological and physiological changes in stem xylem vessel number, ROS-scavenging abilities and drought response compared to control plants. Phylogenetic analysis revealed that hTTP and its homologous proteins are relatively conserved in plants and animals. Our results may be helpful for the understanding of the evolutionary process of homologous proteins in nature.

## Materials and methods

### Plant materials and growth environment

A hybrid poplar (*Populus deltoides* × *P. euramericana* cv 'nanlin895') was used for genetic transformation. All plant materials were grown in the greenhouse under natural light and temperature 22−25 °C.

### Vector construction and plant transformation

The coding region of *hTTP* was inserted into the pCAMBIA1300 vector using the appropriate primers (Forward: 5'-TACGAACGATAGCCATATGGCCAACCGTTACACCATGG-3'; Reverse: 5'-GGCGCGGCCGCAGGCATCTCAGAAACAGAGATGCGATTGAAG-3'). The resulting construct was transformed into *Agrobacterium tumefaciens* strain GV3101 using electroporation. Transformed plants were obtained via the Agrobacterium-mediated leaf disc method, and selected on 1/2 MS medium containing 5 mg·L^−1^ hygromycin. Positive transformants were identified by PCR analysis of genomic DNA with the primers (35Spro-F, 5'-ATGACGCACAATCCCACTATCC-3'; and hTTP-R: 5'-CTCAGAAACAGAGATGCGATTGAAG-3').

### Quantitative Real-Time RT-PCR (qRT-PCR)

Total RNA isolation and first-strand cDNA synthesis were performed as described previously^[14]^. qRT-PCR analysis of *hTTP* expression were conducted on a LightCycler®480 Detection System (Roche) using the primers (Forward: 5'-CCTCGCGCTACAAGACTGAG-3'; Reverse: 5'-GGCCCTGGAGGTAGAACTTG-3'). Each reaction contained 10 µL of SYBR Premix ExTaq II (TaKaRa), 1 µL of diluted cDNA template, 2 µL of primers (a final concentration of 10 nM) and 7 µL of H_2_O. The expression was normalized using reference gene *PdUBQ* (BU879229; the primers, Forward: 5'-GTTGATTTTTGCTGGGAAGC-3'; Reverse: 5'-GATCTTGGCCTTCACGTTGT-3') and determined by the 2^−ΔΔCᴛ^ method^[19]^. Data represent the average of at least three biological replicates.

### Microscopy

Basal stems of 3-month-old transgenic poplar lines (> 3 plants for each line) were sampled for sectioning as described previously^[14]^. Briefly, 0.5-cm stem segments were submerged in 4% paraformaldehyde for 3 d, dehydrated in a graded ethanol series, and finally incubated in pure paraplast. Stems tissues were cut by a Leica RM 2235 microtome (Leica) with a thickness of 7 μm. The sections were stained with Toluidine Blue-O (TBO, 1% w:v) and then observed under an Olympus DX51 microscope (Olympus). For each line, at least 15 locations close to the cambium from five plants were selected for measurement of vessel cell number, and at least 250 vessel cells from five plants were selected for measurement of vessel lumen area using imageJ software.

### Drought treatment

Wild-type (WT) and transgenic poplars were cultivated in suitably sized pots, and each pot had a tray. Three-month-old plants were subjected to a drought stress by withholding water supply for 20 d, and control plants had ≥ 70% soil relative water content (SRWC)^[20]^. Physiological parameters were recorded and measured before and after drought treatment.

### Measurement of physiologic parameters

Physiologic parameters of the control and transgenic lines were measured according to our previous method^[17]^. At the 20^th^ d of drought treatment, the 5^th^ to 7^th^ leaves were selected to detect the net photosynthetic rate, stomatal conductance, transpiration and chlorophyll a+b content using a Li-6400 photosynthesis system (Li Cor Biosciences, Lincoln, NE, USA). The 4^th^ to 6^th^ leaves were selected to detect the contents of malondialdehyde (MDA), hydrogen peroxide (H_2_O_2_), four ROS scavenging enzymes (superoxide dismutase, SOD; peroxidase, POD; ascorbic acid peroxidase, APX and catalase, CAT) and three osmoregulation (proline, soluble protein and soluble sugar). For each genotype, at least three biological replicates were performed.

### Transcriptional activation assays

Full-length *hTTP* and two fragments including its CCCH domain (107-176aa) or excluding the CCCH domain (ΔCCCH domain, 1-106 + 177-332aa) were separately fused with the GAL4 DNA-binding domain in pGBKT7 (Clontech). The fragment with hTTP ΔCCCH domain was synthesized by BGI Genomics (Shenzhen, China). The resulting constructs and pGBKT7 (a negative control) were transformed into yeast strain AH109 cells and cultured on the synthetic dropout medium without tryptophan (SD/-Trp) or without tryptophan, histidine, and adenine (SD/-Trp/-His/-Ade). Transcriptional activity was assessed based on the growth status in SD/-His medium after 3 d. Primers were as follows: hTTP and hTTP ΔCCCH domain (Forward, 5'-ATGGAGGCCGAATTCATGGCCAACCGTTACACCATGG-3' and Reverse, 5'-GATCCCCGGGAATTCACTCAGAAACAGAGATGCGATTGAAG-3'); hTTP CCCH domain (Forward, 5'-ATGGAGGCCGAATTCATGCCCTCGCGCTACAAGACTG-3' and Reverse, 5'-GATCCCCGGGAATTCTTCGCTAGGGTTGTGGATGAAG-3').

### Phylogenetic analysis

hTTP protein was used as query sequence to search the genomes of 13 species, including green algae (*Chlamydomonas reinhardtii*), moss (*Physcomitrium patens*), spike moss (*Selaginella moellendorffii*), Arabidopsis (*Arabidopsis thaliana*), poplar (*Populus trichocarpa*), rice (*Oryza sativa*), soybean (*Glycine max*), yeast (*Saccharomyces cerevisiae*), nematode (*Caenorhabditis elegans*), fruit fly (*Drosophila melanogaster*), frog (*Xenopus laevis*), mouse (*Mus musculus*), and human (*Homo sapiens*) in National Center for Biotechnology Information (NCBI) with the Basic Local Alignment Search Tool algorithm BLASTP and with E-value cutoff set as 1e-005. Multiple sequence alignment of amino acid sequences from these CCCH proteins were performed using the ClustalX (version 1.83) program, and the phylogenetic trees were then generated using the neighbor-joining (NJ) method with 10,000 bootstrap replicates in MEGA 7.0.

### Statistical analysis

Data were presented as mean ± standard errors. The statistical significance of differences between data was evaluated using ANOVA followed by Duncan's multiple range test (*p* < 0.01).

## Results

### Ectopic expression of *hTTP* in poplar inhibits growth and xylem vessel development

**Figure 1:**
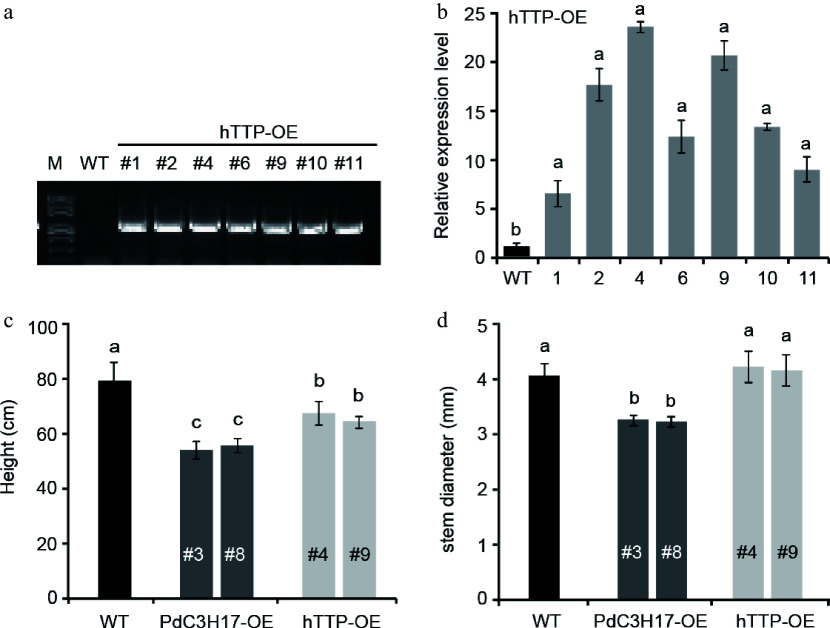
Phenotypes of *hTTP* overexpression poplar lines. (a) PCR identification of representative *hTTP* overexpression (hTTP-OE) poplar lines on genomic DNAs using pro35S and *hTTP*-specific primers. Wild-type (WT) poplar was used as a control. (b) qRT-PCR analysis of *hTTP* expression levels in seven hTTP-OE lines. (c), (d) Height and stem diameter of 3-month-old WT, *PdC3H17* overexpression (PdC3H17-OE), and hTTP-OE lines. In (b)−(d), different lowercase letters above bars indicate significant differences at *p* < 0.01 by ANOVA.

Our previous studies showed that PdC3H17, a homolog of hTTP in poplar, positively regulated cambium division, xylem differentiation and SCW deposition in stem^[14−16]^. Here, we generated transgenic poplar lines overexpressing *hTTP* to investigate whether hTTP functions in poplar. Among seven independent overexpression lines, two lines #4 and #9 were selected for subsequent analysis, because in them the expression levels of *hTTP* were the highest compared with in control plants (Fig. 1a, b). At growth of three months in soil, *hTTP* overexpression lines (hTTP-OE) exhibited 15.1% shorter than wild-type plants, similar to the dwarf phenotype of *PdC3H17* overexpression lines (PdC3H17-OE, the controls) (Fig. 1c). This result suggested that hTTP, like PdC3H17^[14]^, may negatively regulate stem elongation in poplar. Statistic analysis of stem diameter revealed no significant difference between hTTP-OE and WT plants, but thinner in PdC3H17-OE plants than in WT plants (Fig. 1d).

**Figure 2:**
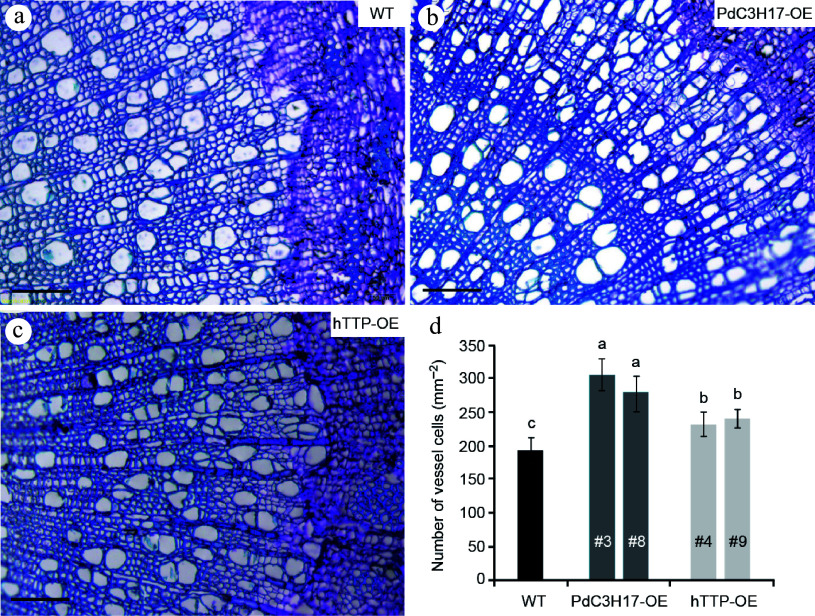
Overexpression of *hTTP* in poplar increases the number of stem xylem vessel cells. (a)−(c) Basal stem sections of 3-month-old WT, PdC3H17-OE and hTTP-OE plants. Bars = 100 um. (d) Statistical analysis of the number of xylem vessel cells in WT, PdC3H17-OE and hTTP-OE plants shown in (a)−(c). For each line, at least 15 locations close to the cambium from five plants were selected for measurement. Data are presented as mean ± SD. Different letters denote significant differences between samples (*p* < 0.01).

To analyze whether *hTTP* overexpression affects the morphology of stem xylem cells, we sectioned basal stems of 3-month-old plants for microscopic observation. The number of vessel element was the greatest in the stems of PdC3H17-OE plants, followed in those of hTTP-OE plants, and the smallest in those of WT plants (Fig. 2). Vessel lumen area of WT, PdC3H17-OE and hTTP-OE plants separately were 3,080.5 ± 178.2, 3,183.5 ± 286.1, and 3,125.2 ± 137.8 um^2^. No significant differences were observed among the three genotypes.

### Ectopic expression of *hTTP* in poplar enhances tolerance to drought stress

**Figure 3:**
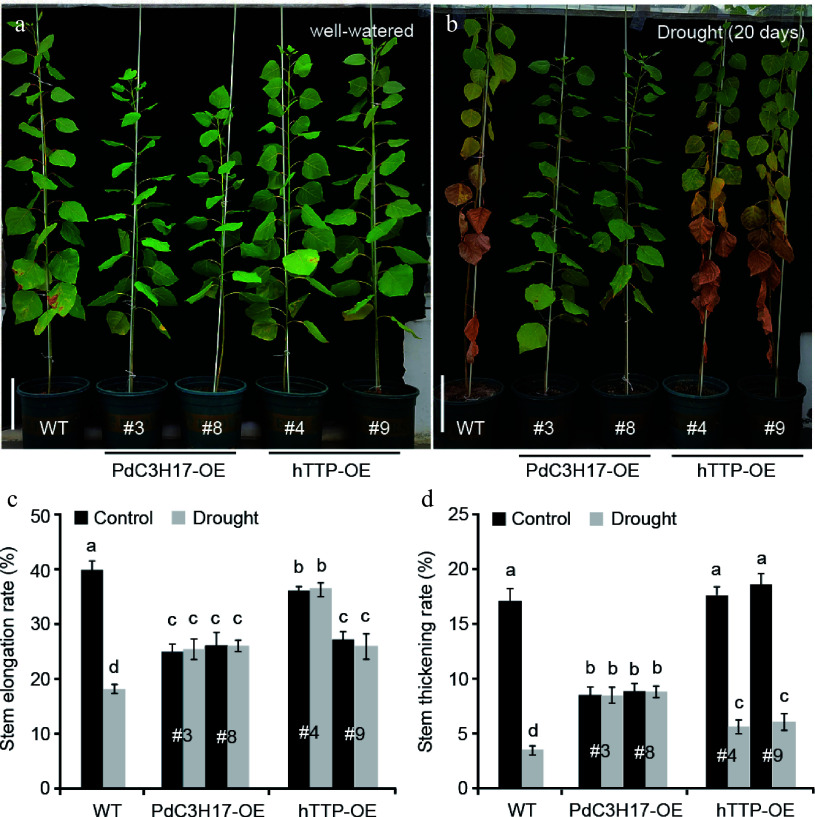
Transgenic poplar lines overexpressing *hTTP* show enhanced drought tolerance. (a), (b) Drought responses of 3-month-old WT, PdC3H17-OE and hTTP-OE poplar lines. Bars = 10 cm in (a). (c), (d) Stem elongation and thickening rates of WT, PdC3H17-OE and hTTP-OE plants shown in (a), (b). For each line, at least five plants were used for measurement. Data are presented as mean ± SD. Different letters indicate significant differences between samples (*p* < 0.01).

The structure and size of xylem vessel cells are key factors affecting water transport and are the important determinants of drought tolerance in plants^[21,22]^. Since ectopic expression of *hTTP* in poplar resulted in an increase in the number of stem xylem vessels (Fig. 2), we further investigated whether hTTP-OE plants are associated with drought stress response, similar to PdC3H17-OE plants^[17]^. Under well-watered conditions, WT plants grew observably faster than hTTP-OE and PdC3H17-OE (control) plants. At the 20^th^ d of withdrawing water, the leaves of WT plants were seriously withered and partially shed, while the leaves of PaC3H17-OE lines became slightly yellow (Fig. 3a, b). hTTP-OE lines displayed stronger resistance to drought stress than WT plants, but weaker resistance than PaC3H17-OE lines. It is well known that drought stress decreases plant height and ground diameter^[23]^. Here, we found that long-term drought stress induced a reduction of stem elongation rate by 55.3% and of stem thickening rate by 82.1% in WT plants (Fig. 3c, d). Compared with WT plants, drought treatment had no effect on stem elongation and stem thickening of PdC3H17-OE plants and had smaller effects on those of hTTP-OE plants. These results indicated that overexpression of *hTTP* in poplar enhances drought resistance.

### Ectopic expression of *hTTP* in poplar affects the photosynthesis under drought stress conditions

**Figure 4:**
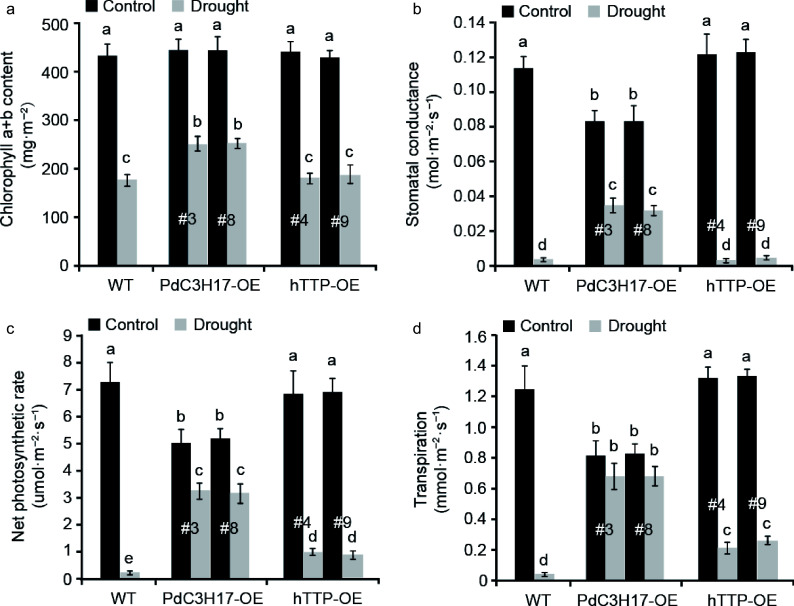
Transgenic poplar lines overexpressing *hTTP* have higher photosynthetic capacities than control plants after drought treatment. (a)−(d) Effects of *hTTP* overexpression in poplar on chlorophyll a+b content, stomatal conductance, net photosynthetic rate and transpiration under drought treatment. For each line, at least five plants were used for measurement. Data are presented as mean ± SD. Different letters indicate significant differences between samples (*p* < 0.01).

Woody plants adjust above- and below-ground traits including photosynthesis to adapt to drought stress^[24]^. In this study, we investigated the photosynthetic capacity in transgenic poplar leaves with or without drought treatment (Fig. 4). Under well-watered conditions, no significant differences for chlorophyll a+b content, stomatal conductance, net photosynthetic rate and transpiration were observed between WT and hTTP-OE plants. In contrast, WT and hTTP-OE plants had higher stomatal conductance, net photosynthetic rate and transpiration than PdC3H17-OE (control) plants. When the plants were treated by drought stress for 20 d, four photosynthetic parameters visibly decreased in all three genotypes, but the decrease rate of WT plants was significantly larger than those of PdC3H17-OE and hTTP-OE plants. These results indicated that transgenic poplars overexpressing *hTTP* may adapt to drought stress by alteration of photosynthetic capacities.

### Ectopic expression of *hTTP* in poplar changes the levels of ROS and osmotic adjustment substances under drought stress condition

**Figure 5:**
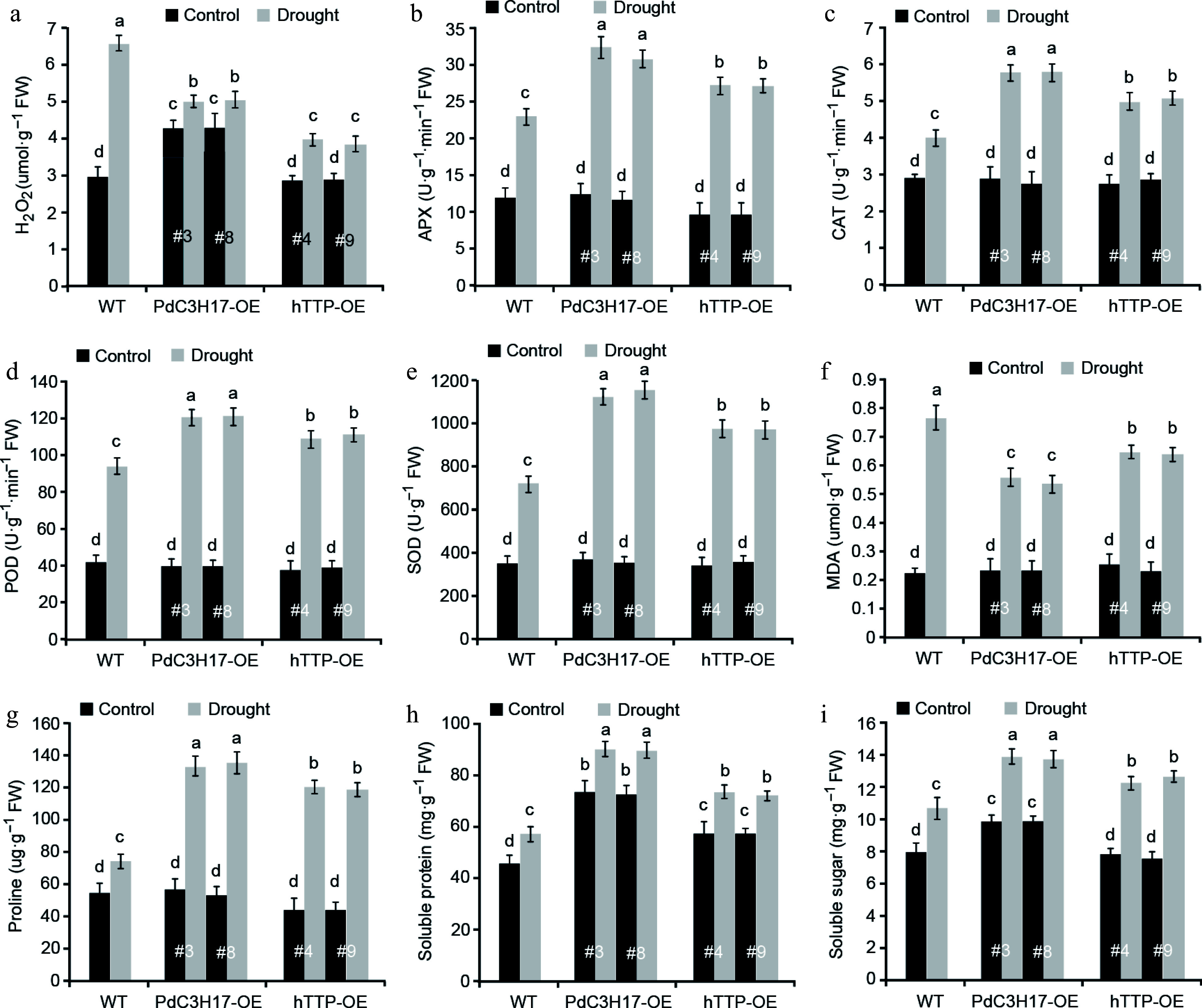
Transgenic poplar lines overexpressing *hTTP* show stronger ROS-scavenging abilities than control plants after drought treatment. H_2_O_2_, hydrogen peroxide. MDA, malondialdehyde. APX, ascorbate peroxidase. CAT, catalase. POD, peroxidase. SOD, superoxide dismutase. For each line, at least five plants were used for measurement. Data are presented as mean ± SD. Different letters indicate significant differences between samples (*p* < 0.01).

We further examined whether *hTTP* overexpression in poplar affects drought-induced accumulation of ROS and osmotic adjustment substances. Hydrogen peroxide (H_2_O_2_) is one of the major ROS that significantly accumulates under stress conditions, leading to oxidative damage in plants^[25]^. As expected, the levels of H_2_O_2_ were increased in WT, hTTP-OE and PdC3H17-OE (control) plants after drought treatment, and such increase was more significant in WT plants than in hTTP-OE and PdC3H17-OE plants (Fig. 5a). Consistent with these, the activities of four antioxidants (APX, CAT, POD and SOD) that can scavenge ROS, showed fewer increase in WT plants compared with hTTP-OE and PdC3H17-OE plants after drought treatment (Fig. 5b−e). Drought treatment induced more accumulation of MDA, an indicator of cytomembrane oxidative damage, in WT plans relative to in hTTP-OE and PdC3H17-OE plants (Fig. 5f), implying that hTTP-OE plants may undergo less membrane damage than WT plants. In addition, the contents of free proline, soluble protein and soluble sugar were more significantly increased in hTTP-OE and PdC3H17-OE plants than in WT plants after drought treatment (Fig. 5g−i). These results indicated that *hTTP* overexpression may enhance the ROS scavenging capacity and thereby confer plant resistance to drought stress.

### hTTP has transcription activation ability in yeast cells

**Figure 6:**
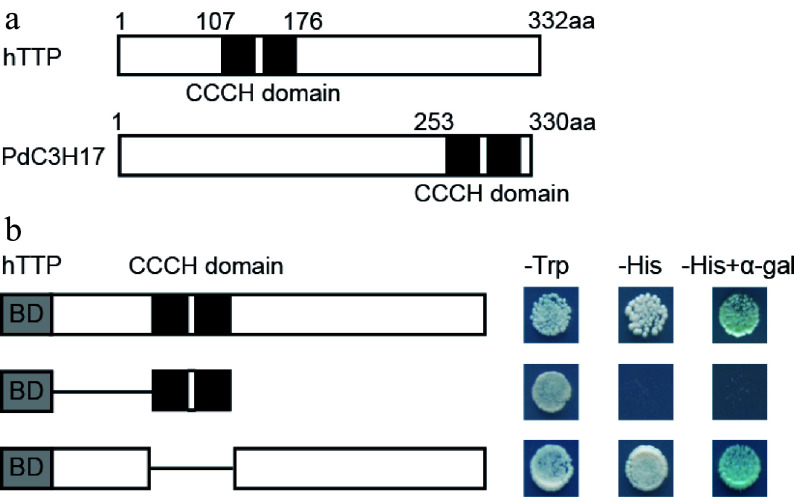
Transcription activation activity of hTTP. (a) Diagram of hTTP and PdC3H17 proteins. (b) GAL4 DNA binding domain fused with full-length or truncated hTTP sequences were expressed in the yeast strain AH109. LacZ activity was observed in SD/-His medium containing X-α-Gal. pGBKT7 was used as a negative control. aa, amino acid.

hTTP protein contains two tandem CCCH zinc-finger RNA-binding domains (C-X_8_-C-X_5_-C-X_3_-H) (Fig. 6a), similar to PdC3H17 protein^[14]^. To examine the transcriptional properties of hTTP in yeast cells, the full-length and selected portions of hTTP were individually fused to the GAL4 DNA-binding domain. Transcription of the *HIS* reporter gene was activated by GAL4-hTTP and GAL4-hTTP ΔCCCH domain, but not by the GAL4-CCCH domain (Fig. 6b). Theses results indicated that the C-terminal sequence of hTTP may possess transcriptional activation capacity.

### Phylogenetic analysis of hTTP and its homologous proteins in plants and animals

**Figure 7:**
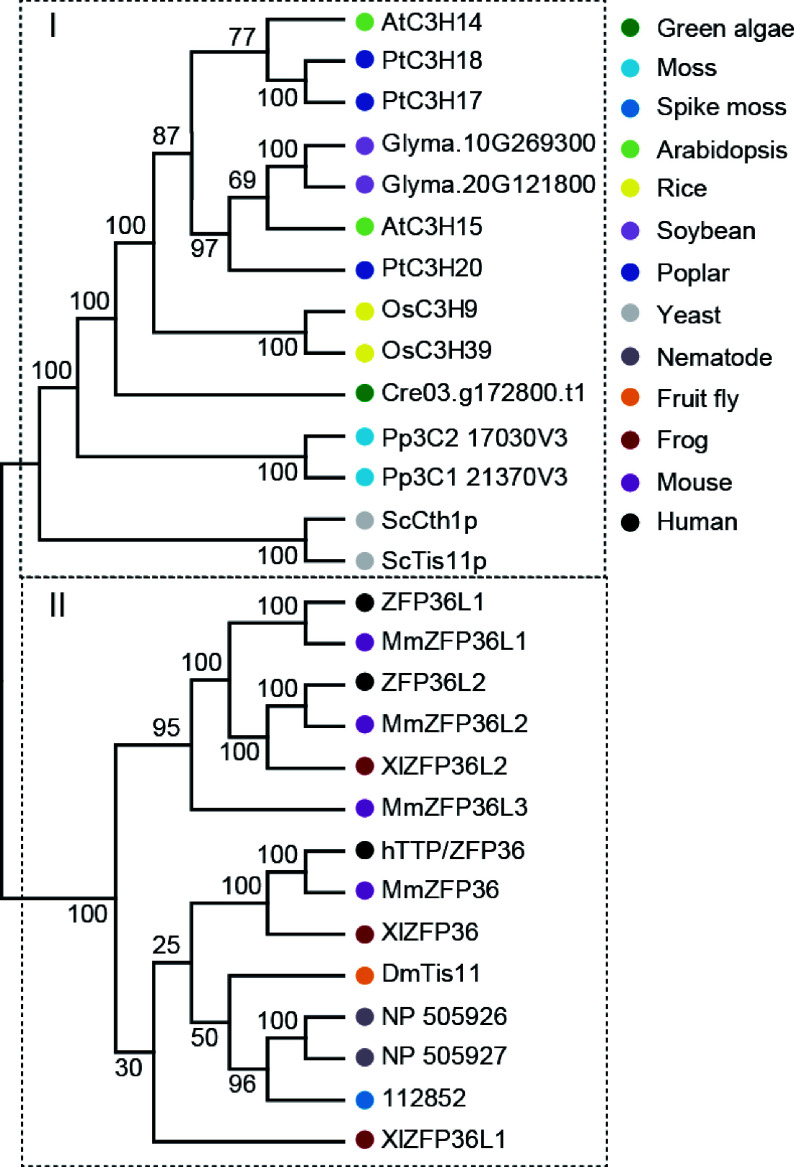
Phylogenetic analysis of hTTP and its homologous proteins from 13 representative species. The neighbor-joining (NJ) tree was constructed based on the alignment of full-length protein sequences. MEGA 7.0 was used with 1,000 bootstrap replicates.

To examine the evolutionary relationship of hTTP family, 28 homologs of hTTP were identified in 13 representative species, including one unicellular fungus (yeast), two primitive plants (green algae and moss), one pteridophyte (spike moss), four angiosperms (Arabidopsis, rice, soybean and poplar), one worm (nematode), one arthropod (fruit fly), one amphibian (frog) and two mammals (mouse and human). An un-rooted phylogenetic tree was constructed based on the alignment of full-length protein sequences using the neighbor-joining method with 10,000 bootstrap replicates. At least one TTP family member was identified in each species, suggesting that the type of CCCH proteins appeared early in the evolution of unicellular fungus, and have persisted in modern eukaryotes. Phylogenetic analysis revealed that the 28 TTP family members were divided two distinct clusters (Fig. 7), indicating a possible functional divergence of hTTP homologs between plants and animals. In particular, counterparts among human, mouse and frog exhibited cross-distribution in one group, suggesting the functional conservation and similarity of these TTP proteins.

## Discussion

Poplar, a fast-growing tree, has been widely planted in China, providing pulp and paper, oriented strand board, chemicals and biofuels. Dissection of poplar gene function and their application to wood improvement are challenging and long-term tasks. Our previous studies showed that the poplar genome contains 91 CCCH members, of which PdC3H17 positively controls cambium division, xylem differentiation, secondary wall formation, and drought response^[7,14−16]^. In this study, we provided evidence showing that ectopic expression of human *TTP* in poplar affected xylem development and drought response, similar to the effects of *PdC3H17* overexpression. This result confirmed that the application of animal genes in plants is effective. Our conclusion is also supported by several other studies. In Arabidopsis, ectopic expression of human *APOLIPOPROTEIN D* (*APOD*) partially rescues the defects of *lcnp*, a mutant line of the Arabidopsis ortholog of *APOD*^[26]^. In rice and potato, ectopic expression of the human RNA demethylase gene *FTO* stimulates root meristem cell proliferation and tiller bud formation, and promotes photosynthetic efficiency and drought tolerance^[27]^. In apple, ectopic expression of the human melatonin synthase gene *HIOMT* enhances plant tolerance against drought stress^[28]^. All these findings highlight the gene regulatory potential of cross species.

Poplar has evolved versatile mechanisms to mitigate drought stress, including altering plant morphology, reducing transpiration, scavenging ROS, and generating abscisic acid (ABA)^[29]^. Of them, photosynthesis is believed to be the primary physiological target^[30]^. Indeed, drought-induced photosynthesis was significantly higher in PdC3H17-OE and hTTP-OE plants than in WT plants. In general, stomatal regulated reduction in transpiration results in photosynthetic inhibition in the stressed leaves. However, PdC3H17-OE and hTTP-OE plants had higher stomatal conductance and transpiration under drought stress. Extensive studies show that stomatal conductance does not solely control the changes of foliar photosynthetic rate. Mesophyll conductance and biochemical limitation are the major non-stomatal factors that modulate the photosynthetic rate^[31,32]^. It is possible that these non-stomatal factors contribute to higher photosynthesis in PdC3H17-OE and hTTP-OE plants under drought stress. Vessel elements of stem xylem are the primary water conducting cells in trees. We found that hTTP-OE plants had more stem xylem vessel cells than WT plants, which may be helpful for increasing hydraulic safety and reducing the risk of embolism, thereby contributing to stronger drought tolerance in transgenic plants. Similarly, AREB1-mediated histone acetylation of PtrNAC6/7/120 significantly influences vessel development and in turn alters drought response in poplar^[21]^. Increasing the ROS scavenging capacity can help plants to cope with drought stress^[33]^. We here showed that overexpression of *hTTP* in poplar reduced drought-induced accumulation of H_2_O_2,_ one of the major ROS, and MDA. Consistently, the activities of four major ROS-scavenging enzymes (APX, CAT, POD, and SOD) increased more in PdC3H17-OE and hTTP-OE plants than those in WT plants after drought treatment. Thus, another possible reason for stronger drought tolerance of hTTP-OE plants is these transgenic poplars had enhanced ROS scavenging capacity after drought stress. In addition to functioning in stress responses, H_2_O_2_ acts as a signaling molecule that promotes xylem cell differentiation and lignification in poplar^[34]^. Higher H_2_O_2_ levels were detected in PdC3H17-OE plants compared with WT plants under normal condition. This result, combined with the finding that PdC3H17 positively regulates xylem differentiation and SCW deposition^[14]^, suggested that H_2_O_2_ signaling may influence xylem development in PdC3H17-OE plants.

Plant TZF proteins control multiple cell processes including cell proliferation and cell differentiation by protein-DNA, protein-RNA and protein-protein interactions, similar to the regulatory mechanisms of animal TZFs^[6]^. Functional characteristics of hTTP and AtC3H14 have demonstrated that the CCCH domains of both proteins are necessary for the degradation of target mRNAs in cytoplasmic foci^[8,35]^. Our current results revealed that hTTP and its homologous proteins in plants are relatively conserved. In particular, hTTP and PdC3H17 have high identify over their CCCH domains^[14]^. The fragment excluding CCCH domain in hTTP possessed transcriptional activation ability in yeast cells, similar to the N-terminal sequence of PdC3H17 that excludes its CCCH domain^[17]^. Thus, it is possible that hTTP functions in poplar by the PdC3H17-mediated pathway at both transcriptional and post-transcriptional levels. This hypothesis needs to be further validated experimentally.

## Conclusions

In the study, we investigated how overexpression of the CCCH protein TTP from human in a hybrid poplar affects xylem development and stress response. Compared with control plants, stem xylem vessel number, and photosynthetic and ROS-scavenging capacities of hTTP-OE plants significantly changed, similar to those of PdC3H17-OE plants. These results suggest that hTTP may function in poplar through the PdC3H17-mediated network.

## Author contributions

The authors confirm contribution to the paper as follows: experiment and drafted the manuscript: Chai G, Zhou G, Zhuang Y; materials preparation and performing the experiment: Zhuang Y, Chen Y; data processing: Wang Q, Chen Y, Yan L; manuscript revision: Li S. All authors reviewed the results and approved the final version of the manuscript.

## Data availability

All data generated or analyzed during this study are included in this published article.
